# Garlic and Hypertension: Efficacy, Mechanism of Action, and Clinical Implications

**DOI:** 10.3390/nu16172895

**Published:** 2024-08-29

**Authors:** Christopher Sleiman, Rose-Mary Daou, Antonio Al Hazzouri, Zahi Hamdan, Hilda E. Ghadieh, Bernard Harbieh, Maya Romani

**Affiliations:** 1 Department of Biomedical Sciences, Faculty of Medicine and Medical Sciences, University of Balamand, Tripoli 1300, Lebanon; christopherj.sleiman@std.balamand.edu.lb (C.S.); rose-mary.daou@std.balamand.edu.lb (R.-M.D.); antonio.hazzouri@std.balamand.edu.lb (A.A.H.); zahi.hamdan@std.balamand.edu.lb (Z.H.); hilda.ghadieh@balamand.edu.lb (H.E.G.); 2 Department of Family Medicine, American University of Beirut, Beirut 1107-2020, Lebanon

**Keywords:** garlic, hypertension, history, complementary and alternative therapy

## Abstract

Hypertension is a major risk factor for heart disease and stroke. Garlic has a long history of use in traditional medicine for various conditions, including hypertension. This narrative review examined the scientific evidence on the efficacy of garlic in lowering blood pressure. It explores the historical uses of garlic in different cultures for medicinal purposes and delves into the phytochemical composition of garlic, highlighting key components, like allicin and ajoene, that are believed to contribute to its potential health benefits. Clinical studies that investigated the effects of garlic and garlic-based supplements on blood pressure are presented, with the findings suggesting that garlic consumption may modestly reduce blood pressure, particularly in individuals with mild hypertension. Potential mechanisms of action include increased nitric oxide production, improved endothelial function, and antioxidant properties. While garlic may offer some benefits for blood pressure management, it should not be considered a substitute for conventional antihypertensive medications. Further large-scale, long-term clinical trials are warranted to establish the efficacy of garlic in managing hypertension, including the optimal dosage and formulation.

## 1. Introduction

The cardiovascular system plays a crucial role in maintaining homeostasis and ensuring the efficient circulation of blood throughout the body. Several studies showed that dysfunction within this system can result in a spectrum of cardiovascular diseases (CVDs), including atherosclerosis, hyperlipidemia, and hypertension [1,2,3,4]. Hypertension, which is a prevalent cardiovascular condition, necessitates ongoing exploration of diverse interventions to manage its impact on global health. Amidst the pharmacological interventions available, there is growing interest in exploring natural remedies, with garlic emerging as a promising candidate [5]. Garlic is the edible bulb from a plant in the lily family, and it is renowned for its culinary uses and traditional medicinal properties. It has long been associated with various health benefits due to its bioactive compounds, such as sulfur-containing compounds, ajoene, alliin, and allicin [6]. The main bioactive chemical constituents in garlic are organosulfur compounds. Alliin is a precursor to other bioactive chemicals. Alliinase hydrolyzes dehydroalanine to produce allyl sulfenic acid. Allicin reduces oxidative stress, enhances mitochondrial function, and influences CVD risk factors [7,8]. 2-Vinyl-4H-1,3-dithiin has anti-proliferative and anti-migratory properties. Diallyl sulfide (DAS), diallyl disulfide (DADS), diallyl trisulfide (DATS), and allyl methyl sulfide (AMS) all contribute to garlic’s health benefits [7]. Garlic lowers blood pressure by a synchronized interplay of biochemical and physiological mechanisms. While the exact pathways are not fully explained, several key mechanisms were proposed based on research findings. Some of the proposed pathophysiologies include vasodilation via nitric oxide (NO) release, enhanced endothelial function, antioxidant properties, inhibition of angiotensin converting enzyme (ACE) activity, sodium and water excretion, and anti-inflammatory effects [9]. Alliin reduces blood triglycerides, boosts HDL cholesterol, and inhibits myocardial lipid accumulation. Allicin prevents vascular calcification, promotes endothelial cell migration, and lowers blood pressure and plasma lipids. AMS lowers fetal gene expression and protects against oxidative stress [7]. It is important to highlight that the efficacy of garlic in lowering blood pressure can vary based on diverse factors such as the type of garlic used (fresh, aged extract, supplements), individual responses, and dosage [10].

This narrative review aimed to investigate the effectiveness of garlic in improving hypertension, with a specific focus on its constituent phytochemicals and their modes of action. It focused on articles published between 2014 and 2024 that were sourced from PubMed. The scope included randomized controlled trials, clinical trials, meta-analyses, and systematic reviews, with some exceptions for particularly relevant studies outside this timeframe. Excluded from this review were studies that focused on other cardiovascular conditions.

## 2. Historical Use of Garlic in Traditional Medicine

Throughout history, many cultures have used garlic for medicinal purposes, particularly in relation to the heart and blood pressure. In ancient Egypt, the Ebers Papyrus prescribed garlic for heart-related issues. It was also implemented in treating tumors, abscesses, malaise, and parasitic or insect infestations [11]. In Greece and Rome, garlic was used to “cleanse the arteries” and linked to cardiovascular health by figures like Hippocrates and Dioscorides [11]. Charaka, the father of Ayurvedic medicine from 3000 B.C., claimed that “garlic maintains the fluidity of blood and strengthens the heart” [12]. In India, Ayurvedic medicine uses garlic to maintain blood fluidity and strengthen the heart.

Garlic is now well known to reduce blood pressure if used appropriately [13] and a diuretic effect was attributed to it, where it is believed that the mobilization of fluid from the extravascular space results in improved cardiovascular function due to garlic treatment. Furthermore, it was thought to improve elevated serum cholesterol, decrease the aggregation of platelets, and protect the vascular endothelium from damage by LDL [11].

## 3. Garlic’s Broader Historical Uses

Beyond its cardiovascular benefits, garlic has a rich history of use in various cultures for a wide range of ailments, including respiratory problems and infections. For digestive issues, garlic was used to treat diarrhea, worm infestations, and stomach problems [11]. Additionally, it was applied externally for conditions such as skin diseases and dandruff [14]. As for infections, it was believed to have antiseptic properties and was used against plagues, cholera, and other infections. The Talmud, which is a Jewish religious text from the second century AD, prescribes garlic to treat parasitic infections and other disorders [11]. Celsius used garlic to cure tuberculosis and fever in the second century [14]. The authors also state that in 1720, the inhabitants of Marseille were saved from the spread of the epidemic of plague by garlic, and in 1858, Louis Pasteur wrote about garlic killing bacteria, such as Helicobacter pylori [14]. The antiseptic properties of garlic were affirmed in the suppression of cholera in 1913, typhoid fever and diphtheria in 1918 in Beirut, and the influenza pandemic in Spain and the influenza epidemic in America during 1917 and 1918 [14]. As for reproductive issues, it was also used to improve male potency [11].

## 4. Traditional Beliefs about Garlic Use

Traditional beliefs surrounding garlic use span ancient civilizations, such as Egypt, Greece, and Rome. In Egypt, it was incorporated into the daily diet, and it was believed to provide strength and productivity to laborers [11,14]. Ancient Greeks used garlic to enhance athletic performance and bolster soldiers’ strength before battles. Romans similarly valued garlic for its perceived strengthening properties, particularly for soldiers and sailors [11]. Dioscorides recommended using garlic as a remedy against snakebites by drinking a mixture of garlic and wine and against mad-dog bites by applying garlic directly onto the wound site [14]. While it was embraced in some cultures, certain European religious sects opposed its consumption. For example, “Garlic either was not permitted or fancied by the upper Brahmin classes, whereas in other cases, it was applied externally to help repair cuts, bruises, and infections” [11]. In various cultures, such as Persian and Serbian folklore, garlic was used in ritualistic practices to ward off evil spirits. Serbs in Sumadija and near the Croatian border believed witches avoided garlic due to its odor [15]. Certain plants, specifically garlic, have been used in ceremonial practices to ward off crop harm and protect people and habitations [15]. Garlic had apotropaic functions in both the Roman and Persian cultures. Persian folklore, including “the Zoroastrians of Yazd,” employed the plant, along with other strong-smelling plants, like onion and asafoetida, because they had the “magical potential to ward off wicked creatures, like jinns, āls, and fairies” [15]. It was believed that virgin girls and pregnant women needed to be protected from “jealous āls and imps” via garlic [15]. In modern times, garlic continues to be used for its medicinal properties, including as a vermifuge in folk medicine and veterinary practice, particularly in Italy [15].

## 5. Attitudes toward Supplementary Herbal Therapy in Hypertension

Despite modern treatments for hypertension, poor blood pressure control remains a significant issue. To effectively manage hypertension, it is recommended to minimize salt intake; engage in regular physical activity; limit alcohol consumption; adopt the Dietary Approaches to Stop Hypertension (DASH) diet; and implement broader lifestyle modifications, such as stress reduction techniques, regulate weight, and address comorbidities, in conjunction with prescribed antihypertensive. However, studies showed that patients with hypertension utilize complementary and alternative therapies, such as herbal remedies and supplements because they do not believe in the efficacy of drugs for blood pressure control and fear the adverse effects of drugs [16]. A study included 173 Turkish-speaking patients over 18 who were diagnosed with hypertension 6 months later to analyze their attitudes toward herbal remedies in hypertension using the Attitude Scale Toward Using Complementary Treatments (ASUCT) [16]. The study demonstrated a significant correlation (*p* < 0.05) between gender, employment position, and mean ASUCT scale scores. The herbal treatments used by hypertension patients included 43.4% pomegranate syrup, 36.4% garlic, and 43.4% quince leaves as the main herbal therapies used [16]. The study found that hypertension patients primarily accessed information about alternative practices through television and social media. This finding supports female patients’ significantly higher mean ASUCT scores. In this study, the education level also impacted the mean ASUCT score. Primary school education resulted in significantly higher mean ASUCT scores compared with university education (*p* < 0.05). The study indicated that hypertension patients chose herbal applications over medical nutrition treatment [16].

## 6. Phytochemical Composition of Garlic

Garlic contains around 2000 biologically active components; in the literature, sulfur-containing compounds were found to play a great pharmacological role [17]. Fresh raw garlic bulbs mainly consist of water (65%) and carbohydrates (28% divided into 85% fructose, 14% glucose, and 1% galactose [18]), in addition to protein (2%), amino acids (1.2%), fiber (1.5%), fatty acids, phenols, trace elements, and sulfur-containing phytoconstituents (2.3%), with alliin being the main one [19,20]. Valuable nutrients include fat-soluble vitamins (A, K, and E), water-soluble vitamins (C and B-complex vitamins: B1, B2, B3, B6, and B8), and minerals (Ca, Fe, Mg, P, K, Na, and Zn) [21].

**Figure 1:**
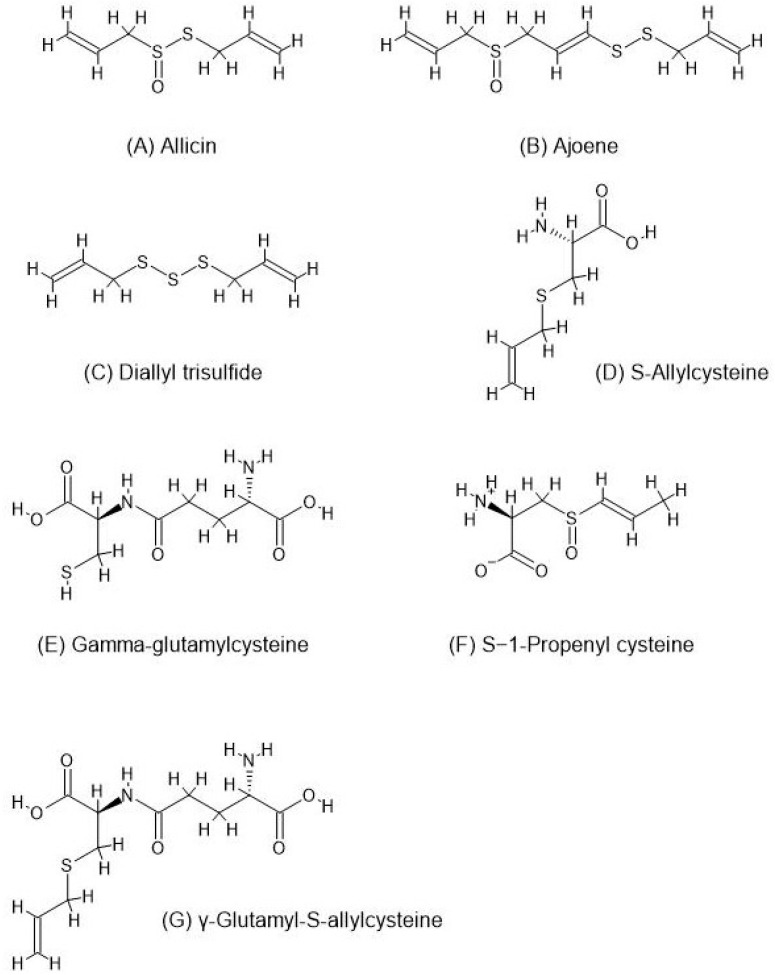
Chemical structures of garlic compounds with antihypertensive effects: (**A**) Allicin, (**B**) Ajoene, (**C**) Diallyl trisulfide (DATS), (**D**) S-Allylcysteine (SAC), (**E**) Gamma-glutamylcysteine (GGC), (**F**) S−1-Propenyl cysteine (S1PC), and (**G**) γ-Glutamyl-S-ally (GSAC).

Upon chopping or crushing garlic cloves, alliin is converted to allicin (Figure 1A) via the activation of alliinase [22,23]. Allicin, which is a prominent constituent of garlic, is unstable and rapidly decomposes into ajoene (Figure 1B), dithiins, allyl methyl trisulfide, diallyl sulfide (DAS), diallyl disulfide (DADS), and diallyl trisulfide (DATS) (Figure 1C) with the addition of heat. The classical garlic odor and taste result from oil-soluble organosulfur compounds (OSDs), like allicin, alliin, and ajoene, rather than water-soluble OSCs. Although the latter—such as S-Allylcysteine (SAC) (Figure 1D), S-allyl mercaptocysteine (SAMC), allyl mercaptan (AM), and allyl methyl sulfide (AMS)—make up the minority of garlic, they show significant properties, such as anticancer activity [19,20]. Additional compounds identified in garlic include steroids, terpenoids, flavonoids, and saponins [18].

Regarding the effects of individual phytochemicals, allicin is ideal because it is hydrophobic and can enter cell membranes without damage or delay; it exerts cardioprotective pharmacological effects, such as antioxidation, anti-inflammation, and anti-apoptosis [8]. First, it induces vasodilation, possibly due to the effect of NO and/or calcium blockage. Second, allicin is probably responsible for inhibiting β-Hydroxy β-methylglutaryl-CoA (HMG-CoA), and thus, lowering total cholesterol and LDL while increasing HDL. Third, cardiac hypertrophy is limited by allicin’s ability to control brain natriuretic peptide (BNP) and inhibit markers responsible for myocyte enlargement. Allicin also blocks reactive oxygen species (ROS), which supports cardiac hypertrophy. Other significant findings include a reduction in hyperglycemia and platelet aggregation [24,25]. Liu et al. (2022) showed that in spontaneous hypertension rats (SHRs), allicin decreased the calmodulin-dependent protein kinase II/Nuclear Factor kappa B (CaMK II/NF-κB) signaling pathway, which results in reduced inflammation and calcium transportation signaling. The net result is a significant inhibition of hypertensive vascular and cardiac modeling [9,26]. Allicin also mediates blood pressure by vasodilation via the nitric oxide (NO), soluble guanylyl cyclase (sGC), cyclic guanosine monophosphate (cGMP), prostacyclin (PGI_2_) adenylyl cyclases (ACs), cyclic adenosine monophosphate (cAMP), and endothelium-derived hyperpolarizing factor (EDHF) pathways [27]. Furthermore, allicin protects the kidneys, heart, and vessels by reducing the angiotensin AT1 receptor (AT1R) and Keap1, thus increasing Nrf2 expression [28]. Recent research showed that allicin pretreatment significantly reduced the increase in serum CK-MB and LDH levels induced by Adriamycin and mitigated the expression of oxidative stress markers, like TNF-α and TGF-β, associated with chemotherapy drugs [8]. In the context of arrhythmias, allicin inhibited the transient outward potassium current (Ito) in mouse ventricular myocytes, with high doses accelerating the voltage-dependent inactivation of Ito, which may contribute to its anti-arrhythmic effects. Additionally, allicin has exhibited an anti-myocardial fibrosis effect in myocardial infarction models by decreasing myocardial collagen deposition and regulating key fibrotic markers, including downregulating collagen I, collagen III, TGF-β1, and Smad3, while upregulating Smad7 [8].

Moreover, another key player in the phytochemical composition is ajoene, (E,Z)-4,5,9-trithiadodeca-1,6,11-triene 9-oxide, which may prevent acute thrombus formation [29]. Synthetic ajoene appears to impose a dose-dependent inhibition of protein prenylation and smooth muscle cell proliferation [30], which pathologically narrow vessel lumens and increase the wall mass due to vascular occlusion, lipid accumulation, and thrombosis, which are features of atherosclerosis and hypertension [31,32]. Overall, ajoene appears to lower blood pressure most effectively when conjugating with other compounds, such as allicin. A recent study that explored garlic for perioperative utility showed that ajoene enhanced the effects of platelet inhibitors, while alliin contributed to hypoglycemia and enhanced insulin sensitivity [25].

Finally, gamma-glutamylcysteine (GGC) (Figure 1E), which is the precursor to glutathione, which is a well-known anti-oxidant [33], is contained in levels up to 0.9% of garlic bulbs and can catabolize into SAC, among other compounds [34]. GGC is a natural ACE inhibitor capable of combining with allicin to dilate arteries and reduce blood pressure [35]. Garlic and garlic-derived organic polysulfides, such as DATS and DADS, act as hydrogen sulfide (H_2_S) donors to induce H_2_S production in a thiol-dependent manner. H_2_S causes vascular smooth muscle cell relaxation, which improves the cardiovascular system [36]. Allicin also releases H_2_S via the action of red blood cells (RBCs) [21]. Additionally, in a study by Dhawan and Jain (2004), essential hypertension patients were given garlic pearls for 2 months. They exhibited a decrease in systolic and diastolic blood pressures, plasma-oxidized low-density lipoproteins (ox-LDL), and plasma and urinary concentrations of 8-iso-Prostaglandin F_2α_ (8-iso-PGF_2α_), while displaying an increase in total antioxidant status (TOS) [37]. These results indicate that garlic reduces vascular dysfunction and hypertension pathogenesis factors. Moreover, the study showed decreased serum total cholesterol, triglycerides, and LDL-C levels, whereas the HDL-C levels remained constant [37]. One mechanism for this is Allicin, which can inhibit squalene-monooxygenase and acetyl-CoA synthetase and, consequently, limit cholesterol production [21].

## 7. Mechanisms of Action

Concerning the antihypertensive effect of garlic and its components, myriad mechanisms of action are discussed throughout the literature, many of which show overlapping molecular pathways. Indeed, the literature focuses on key pathways involving oxidative stress, NF-κB, H_2_S, NO, the renin–angiotensin–aldosterone system (RAAS), and vascular smooth muscle cells (VSMCs). Table 1 provides a summary of the various mechanisms of action of garlic and its constituents in lowering blood pressure.

- Oxidative Stress

SAC can trap ROS, as in male Wistar rats with acute renal failure. Further studies showed that SAC and aged garlic (AG) lowered the activity of nicotinamide adenine dinucleotide phosphate (NADPH) oxidase and increased that of superoxide dismutase in nephrectomy rats in addition to scavenging superoxide (O_2_^−^) and peroxynitrite (ONOO^−^) in vivo, which presumably prevent an increase in systolic blood pressure (SBP) [9]. Allicin effectively scavenges free radicals and traps hydroxyl radicals (OH). It also reduced the Angiotensin II-induced ROS formation in vitro and in vivo by inhibiting NADPH oxidase activity [9].

b. Nuclear Factor Kappa B (NF-κB)

Pyrrolidine dithiocarbamate (PDTC) and SAC suppress NF-κB, which is elevated, along with ROS, in spontaneous hypertensive rats (SHRs). This, along with the effect of PDTC in suppressing cytosolic and mitochondrial ROS, causes a blood pressure reduction in animal models [9].

c. Hydrogen Sulfide (H_2_S)

H_2_S production by the catalytic effect of cystathionine c-lyase (CSE) is enhanced by garlic [9]. Moreover, Benavides et al. showed that human RBCs converted garlic organic polysulfides to H_2_S, which induced vasodilation and reduced blood pressure [36]. Garlic-derived polysulfides are suggested to stimulate H_2_S and enhance NO regulation, which contributes to vasodilation and the consequent blood pressure reduction. Moreover, the consumption of garlic alleviates sulfur deficiency, which may contribute to hypertension [10].

d. Nitric Oxide (NO)

The persistent effect of NO as a vasodilator functions to maintain normal blood pressure [38]. Garlic works similarly to N-acetylcysteine (NAC) by increasing NO bioavailability and reducing the generation of ROS, such as O_2_^−^, which tends to consume NO. In turn, the amount of NO available increases in the presence of garlic or aged garlic extract (AGE) [9]. Notably, garlic no longer forms allicin once boiled or treated with ethanol, and no vasorelaxation occurs [39]. Pedraza-Chaverri et al. (1998) induced hypertension in different rat groups by administering Nω-nitro-L-arginine-methyl-ester (L-NAME), which is a NO synthase blocker. A control group received L-NAME without garlic supplementation and expectedly had increased blood pressure and low urine concentrations of nitrite (NO_2_^−^) and nitrate (NO_3_^−^)—which are anions that reflect NO production. A second group of rats received a combination of L-NAME and garlic supplementation and showed a baseline blood pressure with increased urinary concentrations of NO_2_^−^ and NO_3_^−^. A third group of rats was given garlic supplementation without L-NAME-induced hypertension and similarly showed a baseline blood pressure but with normal urinary anion concentrations, thus verifying the hypothesis that garlic supplementation can block L-NAME-induced hypertension by promoting NO synthesis [40].

Moreover, both SAC and S−1-propenyl cysteine (S1PC) (Figure 1F), which was obtained from the aging of garlic, lowered blood pressure in hypertensive rats. SAC exhibited antioxidant properties, which reduced hypertension in nephrectomized rats, and S1PC modulated molecules such as histidine, tryptophan, and lysophosphatidylcholine. AGE also increased plasma NO and induced the endothelium-dependent vasorelaxation of isolated rat aortic rings. Moreover, allicin was shown to inhibit angiotensin II. γ-glutamyl-S-allylcysteine (GSAC) (Figure 1G) in garlic may also block ACE and induce vasodilation [41]. A recent randomized, triple-blind trial by Serrano et al. (2023) studied the antihypertensive of AGE on subjects with grade I hypertension who are on drug therapy. The study showed a significant reduction in blood pressure via potential mechanisms, including NO release, enhanced antioxidant capacity, reduction of uric acid, and inhibition of ACE. However, there was no increase in H_2_S, no change in endothelial function capacity, and no significant effects on inflammatory markers, although the third could have been due to the low inflammatory status of the volunteers and the low doses of black garlic extract given [42]. From another point of view, a 2020 study on Wistar rats by Hasimun et al. focused on the role of black garlic (BG) on arterial stiffness, as measured by the pulse wave velocity and frontal QRS-T angle. Given that these parameters assess the hypertensive status, the study found that BG increased NO and improved both the arterial status and QRS-T angle [43].

e. Renin–Angiotensin–Aldosterone System (RAAS)

SAC and captopril synergistically inhibit ACE in guinea pigs and decrease blood pressure in rats [44]. Sharifi et al. (2003) treated two-kidney, one-clip (2K-1C) hypertensive rat models with garlic and allicin and noticed a significant reduction in the SBP associated with reduced ACE activity in various organs [45]. Likewise, six garlic-derived peptides, which were identified as Ser-Tyr, Gly-Tyr, Phe-Tyr, Asn-Tyr, Ser-Phe, and Asn-Phe, also inhibited ACE [9]. Further studies on the RAAS system found that garlic and allicin could inhibit epithelial sodium (Na) channels, which resulted in less Na and water being retained and a higher urine Na concentration [9]. Studies showed that garlic reduced sodium-hydrogen exchanger isoform-1 (NHE1) expression in 2K-1C models and prostaglandin E2 (PGE2) in rats; knowing that PGE2 promotes sodium reabsorption at the distal tubule, the net impact of garlic was antihypertensive [9]. Finally, it is noteworthy to mention atrial natriuretic peptide (ANP) when investigating the RAAS. Ajayi and Ajayi (2023) studied the responses of cyclosporine-induced prehypertensive rats to the consumption of garlic powder on angiotensinogen, ACE, and ANP. They observed a significant downregulations in the mRNA expressions of angiotensinogen and ACE after feeding with garlic-based diets for a week, which were likely caused by the increased stimulation and release of natriuretic peptides from the heart; all in all, the study suggests that garlic included in the diet may enhance cardiac cells’ ability to release ANP [46].

f. Vascular Smooth Muscle Cells (VSMCs)

Given that hyperplasia and hypertrophy of VSMCs are involved in the pathogenesis of hypertension via the action of angiotensin II and other signaling molecules, the effect of garlic on VSMCs or angiotensin II is of great interest [47,48]. Studies in spontaneous hypertensive rats showed that garlic, AMS, and DAS interrupted the G_0_/G_1_ cell cycle phase and reduced the extracellular signal-regulated kinase (ERK) phosphorylation; the net outcome was decreased VSMC proliferation. AMS and DAS also prevented Angiotensin II-induced ROS production, which usually contributes to a hypertensive pathology. Likewise, ajoene arrested VSMCs in the G_0_/G_1_ phase and prevented excessive proliferation [9]. The opening of K_ATP_ channels by H_2_S and exogenous H_2_S donors is also a topic of discussion, as potassium ions exit and hyperpolarize the VSMCs to induce systemic vasodilation [6].

**Table 1:** Mechanisms of action of garlic and its constituents in lowering blood pressure.

Biological Factors	Physiologic Effect	Action of Garlic and Its Components
Oxidative stress	Oxidative stress can lead to endothelial dysfunction and the inability to promote vasodilation, fibrinolysis, and anti-aggregation [49].	SAC can trap ROS and lower the activity of NADPH oxidase. AG increases the activity of superoxide dismutase. Allicin scavenges free radicals and traps OH. Both SAC and Allicin reduce Angiotensin II-induced ROS formation [5].
NF-κB	NF-κB can activate genes that produce pro-inflammatory cytokines like tumor necrosis factor-alpha (TNF-α) and interleukin-1 (IL-1). These cytokines can cause inflammation in the blood vessels, which contributes to endothelial dysfunction [50].	Pyrrolidine dithiocarbamate and SAC suppress NF-κB, which is elevated, along with ROS, in SHRs [5].
H_2_S	H_2_S was shown to induce vasodilation and reduce blood pressure [51].	Garlic enhances H2S production by the catalytic effect of CSE [5]. Garlic-derived polysulfides stimulate H_2_S and enhance NO regulation [6,30].
NO	NO is a potent vasodilator and regulator of vascular tone. It helps to maintain low arterial pressure, inhibit platelet aggregation, and prevent smooth muscle cell proliferation [52].	Garlic increases NO bioavailability and reduces the generation of ROS, such as O_2_^−^, which tends to consume NO [5,32,33]. Garlic supplementation can block L-NAME-induced hypertension by promoting NO synthesis [34].
RAAS	The RAAS regulates blood pressure and fluid balance. An overactive RAAS leads to hypertension [53].	SAC and captopril synergistically inhibit ACE in guinea pigs and decrease blood pressure in rats [38]. Garlic and allicin inhibit epithelial Na channels, which leads to less sodium and water retention and a higher urine sodium concentration [5].
VSMCs	VSMCs regulate vascular tone and blood pressure. Changes in the function or phenotype of VSMCs can contribute to the development of hypertension [32].	Garlic, AMS, and DAS interrupt the G0/G1 cell cycle phase and reduce EFK phosphorylation, which leads to decreased VSMC proliferation [5,41,42]. In addition, opening KATP channels by H_2_S and exogenous H_2_S donors induces systemic vasodilation [4].

## 8. Garlic’s Interaction with Antihypertensive Drugs as a Co-Administered Therapy

Numerous clinical investigations demonstrated that garlic (*Allium sativum*) lowers blood pressure, even in a dose-dependent manner. This action is mainly attributable to organosulfur compounds, such as allicin and diallyl disulfide [54]. Allicin boosts erythrocyte GSH levels and promotes the expression of antioxidant enzymes, like SOD, CAT, and GPX, thus reducing oxidative stress and protecting endothelial cells and cardiomyocytes from apoptosis [54]. Additionally, allicin promotes vasodilation by boosting NO release, raising cyclic AMP and GMP levels, and reducing LDL-cholesterol uptake by macrophages in the artery wall, and thus, preventing atherosclerosis [54].

Garlic-derived protein hydrolysate inhibits angiotensin-converting enzyme (ACE) activity and reduces angiotensin II concentrations, acting synergistically with ACE inhibitors and angiotensin receptor blockers [54]. In rats, diallyl trisulfide (DATS), which is a garlic-derived component, significantly increased the oral bioavailability of nifedipine, which was likely due to its influence on the intestinal metabolism of nifedipine. Therefore, caution is advised when using diallyl trisulfide supplements with nifedipine to avoid potentially dangerous plasma concentrations [54].

Garlic supplements interact with hypertension drugs, such as losartan, valsartan, furosemide, and hydrochlorothiazide, which results in complex dynamics in intestinal absorption and plasma concentrations. These interactions may affect the bioavailability of certain medicines, including valsartan and losartan, due to their substantial liver metabolism and enterohepatic cycling, which may reduce intestine absorption, lessen the percentage of dosage absorbed (Fabs), and affect plasma concentrations [54].

Herb–drug interactions can benefit the body, like those between carvedilol and garlic. A study on rats found that combining aged garlic extract (AGE) or S-allyl-L-cysteine (SAC), which is an active component of AGE, with carvedilol had a pathophysiological influence on isoproterenol-induced cardiac dysfunction. Administering high doses of AGE and SAC for 3 weeks, either alone or with carvedilol, significantly reduced serum lactate dehydrogenase (LDH) and creatine kinase MB (CK-MB) activities and increased endogenous antioxidants in heart tissue. This suggests that combining AGE and SAC with carvedilol can prevent structural and functional damage to the myocardium [54].

Combining atenolol with AGE or SAC improves cardioprotection by preserving membrane integrity and reducing oxidative stress [54]. Atenolol acts as a β-blocker, which reduces the cardiac workload, while AGE and SAC provide antioxidant effects and workload reduction. The combination reduces SOD levels and TBARS, which results in less oxidative damage and better protection against isoproterenol-induced cardiac injury. Garlic co-administration with propranolol can improve patient outcomes [54]. Animal studies showed that giving rats garlic increases the bioavailability and half-life of propranolol. Benefits of this include lower blood pressure, cholesterol, triglycerides, and glucose levels, as well as reduced fluid consumption and body weight in hypertensive animals [54].

## 9. Clinical Studies on Garlic and Hypertension

Garlic shows promise for promoting cardiovascular health, especially in managing blood pressure. Garlic and garlic-based supplements help to manage hypertension and related risk factors, such as atherosclerosis, hyperlipidemia, thrombosis, and diabetes [17].

- Blood Pressure Reduction

Results from studies that used garlic supplements suggest their potential to lower blood pressure, especially in individuals with mild hypertension. AGE appears to be as effective as some medications for treating uncontrolled hypertension [42,55]. In addition, it significantly lowered arterial stiffness, pulse pressure, central blood pressure, pulse wave velocity, and gut microbiota [56]. Garlic supplements reduced the SBP by an average of 8.3 ± 1.9 mmHg and diastolic blood pressure (DBP) by 5.5 ± 1.9 mmHg, which is similar to typical anti-hypertensive drugs, according to a meta-analysis of 12 studies that involved 553 hypertensive participants. This reduction was linked to a 16–40% lower risk of cardiovascular events [56].

Research suggests a dose-dependent effect, with higher doses and longer durations potentially leading to greater reductions. Two studies looked at various levels of garlic consumption. In the first experiment, daily supplements of 240 mg, 480 mg, and 960 mg of garlic were given for 12 weeks. At week 12, 480 mg of garlic caused the strongest antihypertensive effects. In a different study, 11 patients received daily supplements of 300, 600, 900, 1200, and 1500 mg of garlic for 24 weeks. Higher doses and longer durations led to more noticeable SBP reductions. However, the dose–response relationship and duration were only the subject of two articles, and the supplements utilized in the two experiments varied. Since it was improper to combine the data, only the dose and duration effects on the SBP from the two trials were taken into consideration [57]. As for the hypertensive effects, garlic supplementation was linked to a substantial drop in the DBP, but only a minor decline in the SBP in nine randomized controlled trials (RCTs) with 423 individuals [58]. For patients with nonalcoholic fatty liver disease (NAFLD), taking supplements containing garlic may be a safe and effective adjunctive treatment to lower blood pressure and lower their risk of cardiovascular disease [59].

b. Cholesterol Impact

Garlic may have a modest impact on cholesterol by potentially lowering triglycerides and LDL cholesterol, while HDL cholesterol requires a longer time to be reduced [58]. According to the quantitative analysis of 999 individuals undertaken by Fu et al. (2023), garlic intake partially modified the serum lipid profile (TG, TGL, HDL), hypertension (SBP), and demographic factors {waist circumference (WC), body mass index (BMI)} of metabolic syndrome (MetS). Moreover, raw garlic and AG may be more efficient than produced garlic for their considerable effects on TC, LDL, SBP, and DBP, as well as the elevation of HDL. Thus, garlic is a food product that may be useful for treating MetS. Another meta-analysis supports the hypothesis that garlic has potential therapeutic benefits as an anti-inflammatory, anti-hypertensive, anti-hyperglycemic, and anti-hyperlipidemic agent [60]. Likewise, a randomized, double-blind clinical trial assessed the effectiveness of freeze-dried garlic extract on blood pressure and lipid profiles in prehypertensive individuals [61]. Participants took two capsules of freeze-dried garlic extract daily for eight weeks. The results revealed a significant reduction in systolic blood pressure (SBP), diastolic blood pressure (DBP) (*p* < 0.001), and mean arterial pressure (MAP) (*p* < 0.001) in the garlic group compared with the placebo group. Additionally, there was a notable decrease in triglycerides (TGs) (*p* < 0.001), low-density lipoprotein (LDL) (*p* < 0.001), and total cholesterol (TC) (*p* < 0.001), while the high-density lipoprotein (HDL) levels increased (*p* < 0.001) [61]. Garlic supplementation also led to a significant rise in blood nitric oxide (NO) levels (*p* < 0.001). The study demonstrated that garlic supplementation effectively lowered blood pressure, improved lipid profiles, and increased nitric oxide levels in prehypertensive individuals [61].

## 10. Studies on Specific Garlic Formulations

Research has explored how different garlic formulations might affect blood pressure.

- Aged Black Garlic Extract

A study showed promise for this extract combined with low-dose SAC in lowering blood pressure for individuals with grade I hypertension and already on medication [42]. A trial that used AGE over 12 weeks showed similar findings in individuals with an SBP ≥ 140 mmHg. AGE supplementation was typically well tolerated, and the degree of blood pressure decrease obtained was equivalent to that of common antihypertensive medications after 12 weeks. In contrast, there was no discernible difference in the treatment groups for the patient subgroup with a baseline SBP of less than 140 mmHg. In addition, Kyolic AGE improved the gut microbiota, as demonstrated by a higher microbial richness and diversity, with a notable increase in the numbers of *Lactobacillus* and *Clostridia* species found after three months of supplementation [56].

b. Time-Released Garlic Powder Tablets

Compared with regular garlic pills, time-released formulations may offer improved absorption and effectiveness in treating mild-to-moderate hypertension [62]. A meta-analysis was done to evaluate the influence of the manufacturing process on garlic composition. It was shown that garlic powder decreased blood TC and LDL-C by approximately 0.41 mmol/L (15.83 mg/dL) and approximately 0.21 mmol/L (8.11 mg/dL). A mean difference of −0.96 mmol/L (−17.30 mg/dL) was observed in lowering fasting blood glucose (FBG) levels. The garlic supplementation group similarly showed decreases of 4.34 mmHg and −2.36 mmHg, indicating a reduction in the SBP and DBP, respectively. Therefore, consistent evidence from this meta-analysis showed that consuming garlic powder lowered the risk factors for CVD, including TC, LDL-C, FBG, and BP [63].

c. Raw Crushed Garlic

WC, SBP, DBP, TG, FBG, and HDL were among the metabolic syndrome components that raw crushed garlic significantly reduced. After taking raw, crushed garlic for four weeks, there was no discernible change in the BMI of patients with MetS. Because raw, crushed garlic has positive effects on MetS components, it can be utilized as a supplemental treatment for people with MetS, both in prevention and treatment [64].

## 11. Garlic-Protein-Derived Novel Angiotensin I-Converting Enzyme Inhibitory Peptides from MDR and HDCF

- Introduction and Mechanism of Action

The in silico screening methods consist of two peptides extracted from the hydrolysis of garlic proteins: MDR and HDCF. First, single or mixed proteases are used to simulate hydrolysis in the in silico screening methods. This is followed by molecular docking and an absorption, distribution, metabolism, excretion, and toxicity (ADMET) analysis in the screening process. Two novel non-competitive ACE inhibitors (TTW and VHW) were identified from simulated hydrolysates via LibDock and structure–activity connections between ACE inhibitory peptides and receptors following the virtual digestion of Chlorella vulgaris proteins by pepsin, trypsin, and chymotrypsin. Compared with other reported ACE inhibitors derived from C. vulgaris, they demonstrated higher ACE inhibition ability in vitro and antihypertensive effectiveness in animals [65]. Because of its many benefits, including its time-, money-, and environmental-saving qualities, this in silico method thus exhibits significant promise in discovering and identifying new bioactive peptides.

According to the study, the tripeptide MGR and tetrapeptide HDCF exhibited the strongest inhibitions of ACE activity, with IC50 values of 26.38 μM and 4.50 μM, respectively. The potent ACE inhibitory effect of HDCF and MGR may be attributed to their C-terminal aromatic amino acids and arginine. The inability of short-chain peptides to effectively bind and interact with ACE may explain why they did not always exhibit greater ACE inhibitory activity than long-chain peptides. After simulated hydrolysis and in silico screening, two new peptides (HDCF and MGR) with the highest ACE inhibitory activities were discovered [65]. The traditional hydrolysis, purification, and characterization process was used to verify that this in silico method was feasible [65].

The study examined the mechanism behind ligand–receptor interactions by utilizing the CDOCKER program to dock MGR and HDCF into the binding pocket of ACE at the lowest binding energy. It was found that MGR and HDCF could interact with the ACE active catalytic site and bind to important amino acid residues, which created a hydrophobic pocket encircled by a hydrophobic electron cloud [65]. Hydrogen bonds were primarily responsible for the binding and interaction of ACE inhibitory peptides with the ACE active site. Additionally, MGR and HDCF established non-bonded contacts with Zn^2+^, which obstructed ACE’s ability to bind to Zn and reduced its biological activity [65].

b. Effect on Body Weight and Organ Coefficients

During 7 weeks of continuous administration of HDCF and MGR peptides, spontaneously hypertensive rats (SHRs) and normotensive WKY rats showed increased body weight. The high-dose administration of these peptides did not significantly affect the growth of normal rats. In SHRs, weight changes were similar regardless of the peptide treatment. However, the kidney, heart, liver, and spleen coefficients were lowered in SHRs treated with HDCF and MGR, suggesting an amelioration of pathological features [65].

c. Effect on Blood Pressure

Administration of the peptides resulted in a dose-dependent reduction of abnormal blood pressure values (SBP and DBP) in SHRs, with a reduction in SBP comparable with a captopril treatment. The long-term administration of high-dose MGR and HDCF significantly reduced the SBP and DBP, indicating their potential as functional foods for blood pressure regulation. Neither MGR nor HDCF affected the blood pressure in normotensive WKY rats, indicating their safety for normal rats. These garlic-protein-derived peptides modulate the renin–angiotensin system (RAS) by suppressing ACE and AT1 receptor expression, with HDCF showing stronger antihypertensive effects than MGR. They restore RAS balance by upregulating the ACE2-Ang(1–7)-Mas axis and downregulating the renin and ACE-AngII-AT1 axis to achieve effects comparable with captopril. Western blotting confirmed reduced renin expression due to peptide administration [65].

High-dose HDCF showed the most significant improvement in cardiac function by reducing CK, CK-MB, and LDH-L levels. H&E staining revealed an improved morphological structure in the hearts of SHRs after peptide intervention [65]. HDCF intervention at doses of 30 and 50 mg/kg body weight effectively increased the eNOS levels in SHRs (9.78%–59.56% increase). High-dose HDCF achieved eNOS levels similar to the normal group, which indicates its potential for lowering blood pressure by improving endothelial function, while MGR did not mitigate the eNOS depletion [65].

## 12. Additional Considerations and Limitations

- Long-Term Effects

More research is needed to understand the impact of garlic on long-term cardiovascular outcomes, such as mortality [5].

b. Garlic Form Matters

Raw and AG may be more effective than processed garlic for lowering elevated blood pressure and managing MetS [58].

c. Dosage and Delivery

Time-released formulations may improve absorption [62]. In a systematic review, meta-analysis, and meta-regression of RCTs, garlic administration’s effects on MetS components were studied. Regarding garlic tablets, 84 men with mild-to-severe arterial hypertension participated in a double-blind, placebo-controlled trial with an active control arm to assess the hypotensive effects of time-released garlic powder tablets (Allicor) compared with ordinary garlic pills (Kwai). The extended action of Allicor, which allows for a higher absorption of the vasoactive components of garlic powder, caused it to be superior to normal garlic supplements for treating mild and moderate arterial hypertension [62].

d. Drug Interactions

Saquinavir for HIV treatment, warfarin, and vitamin B12 [56].

e. Importance of Patient Education

Although garlic has some potential, it does not lower blood pressure rapidly; hence, educational programs can help patients set realistic expectations [66]. While garlic shows promise as a complementary therapy for cardiovascular health, especially blood pressure management, further research is needed to confirm the long-term benefits and optimal usage [67].

## 13. Allergies and Adverse Effects

- Garlic Allergy

Despite the complementary benefits of using garlic in lowering blood pressure as an adjunct therapy, many drawbacks are mentioned in different studies. One study tested using garlic and onion extracts in an observational cross-sectional study. Garlic and onions were among the 44 aeroallergens and meals examined using the prick test and specific IgE determination. Oral provocation and contact tests were conducted in case of a suspected delayed reaction. A total of 356,798 skin tests and 4254 specific IgE determinations were conducted. Of the 8109 patients tested, 2508 (30.92%) presented with symptoms associated with food intake. Food hypersensitivity was detected by a skin test, positive specific IgE, or provocation in 924 patients and was caused by garlic or onions in 27, indicating a prevalence of 2.92%. Immunodetection showed an association between the symptoms and a specific LTP to these bulbs, without cross-reactivity with other LTPs in the Mediterranean diet (peach, wheat). Therefore, allergic hypersensitivity to garlic and onions should not be underestimated and, given their high consumption, should be included in the diagnostic food allergy category [68].

b. Garlic-Induced Gastroenteritis and Esophagitis

Four published case reports of garlic-induced esophagitis and one suspected gastroenteritis were found during a review. In three cases, direct damage resulted from mechanical or caustic effects. In the remaining two cases, garlic was assumed to be the source of eosinophilic inflammation; these cases also included a considerable history of atopic medical conditions. Clinicians should be aware of the possibility of gastrointestinal problems associated with garlic because it is widely used in culinary and medicinal contexts. When considering the differential diagnosis of upper gastrointestinal symptoms in garlic users, particularly in atopic individuals, esophagitis and gastroenteritis should be considered. Comprehensive medical histories, endoscopy, biopsies, and skin tests are all helpful procedures that should be used when necessary in questionable patients [69].

c. Safety and Potential Toxicity Related to Sulfoxide Compounds

Banerjee et al. studied the long-term effects of a low-dose garlic extract in an animal model using endogenous antioxidant enzymes and lipid peroxidation in the liver and kidney. For thirty days, rats were given three doses of fresh garlic homogenate daily (250, 500, and 1000 mg/kg per day). Both light microscopy and ultrastructural analysis of the liver revealed morphological alterations in the animals treated with 1000 mg/kg daily. Histologically, the liver displayed localized and non-specific hepatocyte damage. The sulfoxides found in garlic extract may cause such damage because they can spontaneously exchange with the -SH groups of proteins and enzymes in the body at physiological pH and temperature, which limits their activity [70]. In addition, the same review showed that rats given large dosages (5 mL garlic juice/kg) intraperitoneally and orally experienced weight loss, as well as hepatic and pulmonary toxicity. In one study, hypertensive rats given garlic supplements four times a day experienced irregular pulses, dehydration, weight loss, and lethargic behavior [70]. Overall, the US Food and Drug Administration lists garlic as safe for consumption in humans, although people with hypersensitivity may experience gastrointestinal irritation if they take large amounts of garlic. Heartburn, nausea, vomiting, diarrhea, flatulence, bloating, moderate orthostatic hypotension, flushing, tachycardia, headache, sleeplessness, perspiration, disorientation, halitosis, and an overpowering body smell were among the adverse effects reported in randomized controlled studies [25,70].

Moreover, garlic bulbs’ low protein content and stability limit their future use. Therefore, heterologous expression methods and further research on bioactive peptides in garlic and spice plants are recommended to produce more peptides and to increase their stability [71]. Additionally, garlic can elevate the risk of bleeding, particularly when used in conjunction with other medications that affect platelet aggregation. It can also increase the likelihood of epidural hematoma [25].

## 14. Conclusions

In conclusion, garlic has historically been utilized for its remedial effects by ancient and traditional practices with no awareness of the exact pathophysiological mechanism through which garlic instilled its anti-hypertensive effects. Given the global prevalence of hypertension, it is important to explore evidence-based treatment modalities, including both pharmacological and natural remedies, especially those that are accessible, safe, and effective. Apparently, garlic is legitimately used to lower hypertension through its rich phytochemical profile. More specifically, the bioactive constituents exert antihypertensive effects through multiple mechanisms involving oxidative stress, NF-κB, H2S, NO, RAAS, and VSMCs. These mechanisms collectively contribute to the ability of garlic to reduce blood pressure and improve cardiovascular health. However, further research is needed to elucidate the complex interactions and molecular pathways involved in the antihypertensive effects of garlic. In addition, further studies are warranted to optimize and personalize the form and dosage of garlic in clinical settings. The transition from a historical remedy to evidence-based treatment is extensive and complex, but the ubiquity of garlic enhances its viability as a subject of study.

## Data Availability

Not applicable.
